# Differences in regulation of tight junctions and cell morphology between VHL mutations from disease subtypes

**DOI:** 10.1186/1471-2407-9-229

**Published:** 2009-07-14

**Authors:** Valentina Bangiyeva, Ava Rosenbloom, Ashlynn E Alexander, Bella Isanova, Timothy Popko, Alan R Schoenfeld

**Affiliations:** 1 Department of Biology, Adelphi University, Garden City, NY 11530-0701, USA

## Abstract

**Background:**

In von Hippel-Lindau (VHL) disease, germline mutations in the VHL tumor suppressor gene cause clear cell renal carcinomas, hemangioblastomas, and pheochromocytomas. The VHL gene product is part of an ubiquitin E3 ligase complex and hypoxia-inducible factor alpha (HIF-α) is a key substrate, although additional VHL functions have been described. A genotype-phenotype relationship exists in VHL disease such that specific VHL mutations elicit certain subsets of these tumors. Here, we examine VHL genotype-phenotype correlations at the cellular level, focusing on the regulation of tight junctions and cell morphology.

**Methods:**

Wild-type and various mutant VHL proteins representing VHL disease subtypes were stably expressed in 3 VHL-negative renal carcinoma cell lines. Using these cell lines, the roles of various VHL-associated cellular functions in regulation of cell morphology were investigated.

**Results:**

As a whole, type 1 mutants varied greatly from type 2 mutants, demonstrating high levels of HIF-2α, cyclin D1 and α5 integrin, lower p27 levels, and a spindly, fibroblastic cellular appearance. Type 2 mutations demonstrated an epithelial morphology similar to wild-type VHL in the majority of the renal cell lines used. Knockdown of p27 in cells with wild-type VHL led to perturbations of both epithelial morphology and ZO-1 localization to tight junctions. ZO-1 localization correlated well with VHL disease subtypes, with greater mislocalization observed for genotypes associated with a higher risk of renal carcinoma. HIF-2α knockdown in 786-O partially restored ZO-1 localization, but did not restore an epithelial morphology.

**Conclusion:**

VHL has both HIF-α dependent and HIF-α independent functions in regulating tight junctions and cell morphology that likely impact the clinical phenotypes seen in VHL disease.

## Background

von Hippel-Lindau (VHL) disease is a relatively rare family cancer syndrome, affecting one in 36,000 people [1]. Individuals afflicted with von Hippel-Lindau disease are predisposed to different subsets of tumors, including pheochromocytomas, hemangioblastomas, and renal cell carcinomas (reviewed in [2]). Inactivation of the VHL tumor suppressor gene is responsible for both the hereditary tumors in VHL disease as well as sporadic renal carcinomas and hemangioblastomas [3-8], (reviewed in [9]).

The VHL tumor suppressor gene resides on chromosome 3p25 and consists of three exons (reviewed in [10]). The VHL gene product exists in two major isoforms: full length pVHL_30 _and the smaller pVHL_19_, which results from internal translation of the VHL mRNA from an in-frame start codon [11-13]. Reintroduction of either VHL product into VHL null cells inhibits *in vivo *tumor formation, signifying that both contain tumor suppressor functions [11-13].

VHL gene products (collectively called pVHL) are part of an ubiquitin E3 ligase complex, together with elongin B, elongin C, cullin 2 (Cul2), and Rbx1, that covalently attaches ubiquitin on substrates, targeting them for proteasomal degradation [14-19]. One target substrate that has been overwhelmingly confirmed for the pVHL E3 ubiquitin ligase complex is hypoxia-inducible factor alpha (HIF-α) [20-23]. HIF-α is stabilized in low oxygen environments and forms a heterodimer with HIF-β, which binds to hypoxia-responsive elements in the promoters of hypoxia-inducible genes (reviewed in [24]). The presence of oxygen is required for the hydroxylation of key proline residues on HIF-α, which allows for its recognition by pVHL and its subsequent ubiquitination [25-27].

VHL disease has a strong genotype-phenotype correlation and specific mutations associated with VHL disease have been categorized by clinical subtypes of incidence of specific tumor types (reviewed in [2]) (see Table 1). Type 1 VHL mutations are mostly deletions and truncation mutations that drastically alter pVHL and phenotypically manifest in a low occurrence of pheochromocytoma and high frequency of renal cell carcinoma [28,29]. Type 2 mutations are commonly missense mutations and manifest in a high occurrence of pheochromocytoma. Subtypes of type 2 VHL disease depend on incidence of other tumors, namely hemangioblastomas and renal cell carcinoma (RCC). Type 2A disease is associated with a high incidence of pheochromocytoma, hemangioblastoma, and low incidence of RCC. Type 2B disease exhibits a high incidence of pheochromocytoma, hemangioblastoma, and RCC. Type 2C disease has pheochromocytoma, but not hemangioblastoma and RCC. Disparities among the pVHL mutants in their ability to ubiquitinate HIF-α (through deficiencies either in HIF-α binding or in ability to form a functional E3 ligase complex) have been suggested to underlie the differences in tumor risks among the VHL disease subtypes [22,30-32]. Accordingly, there has been support for the notion that both the higher RCC incidence seen with type 2B and type 1 VHL mutations and the lack of pheochromocytoma in type 1 mutants may be a result of higher HIF-α levels [33,34].

**Table 1:** VHL mutant constructs used in this study

		Clinical phenotype (risk of tumor type)			
			
Mutant VHL	Subtype	Pheo	Hemangio	RCC	References
del 114–178	1	-	++	++	[11,53]
RC161/2QW	1				[53]
S65W	1				[29,58,59]
N78S	1				[28,29]
L158P	1				[29]
L188Q	1				[29]^a^

Y98H	2A	++	++	-	[29,60,61]
Y112H	2A				[28,29,62]

Y98N	2B	++	++	++	[4,63]^a^
Y112N	2B				[63]
R167Q	2B				[29,58,64,65]
R167W	2B				[28,29,58,64,65]

V84L	2C	++	-	-	[66]^a^
L188V	2C				[28,61,67]

Pheo, pheochromocytoma; hemangio, hemangioblastoma; RCC, renal cell carcinoma

^a ^subtype designation based on a small family

There is increasing evidence that ubiquitination of HIF-α is necessary for VHL-dependent suppression of tumor progression [35-38]. However, there are also several functions related to VHL, such as assembly of extracellular matrix, formation of β1-integrin adhesions, regulation of integrin levels, stability of microtubules, organization of intercellular junctions, maintenance of cellular polarity and cell morphology, cell differentiation, and inhibition of autophagy, for which the relationship to HIF are either currently unclear or may be independent of HIF-α ubiquitination/degradation [39-49]. Along these lines, nonfunctional VHL has been shown to disrupt intercellular junctions in an HIF-independent manner [44]. However, other studies have indicated that such intracellular junctions may be influenced by HIF and/or hypoxia [50,51], indicating a need for clarification of the role of HIF in this VHL-associated phenotype.

In this study, we introduced a spectrum of VHL mutant constructs covering the range of disease phenotypes into several VHL-null renal cell lines and focused on the effects of these mutations on tight junction formation and cell morphological changes. Various VHL target proteins were assessed to determine their roles in these VHL-dependent morphology changes. We report here that there was a stark contrast between type 1 and type 2 VHL mutants in their HIF-α status, levels of integrins and cell cycle proteins, tight junctions, and cell morphology. In particular, loss of VHL roles in suppression of integrin levels and regulation of tight junction formation correlated with VHL disease subtypes and are likely to play a part in initiation of renal tumorigenesis.

## Methods

### Cell lines and cell culture

Renal cell lines (293T, 786-O, and A498) were obtained from the American Type Culture Collection, whereas RCC10 renal carcinoma cells were provided by Miguel Esteban (Imperial College, London). All cells were grown in Dulbecco's modified Eagle's medium containing 10% Serum Supreme (BioWhitaker) and penicillin-streptomycin (100 U/ml and 10 μg/ml, respectively). 786-O cells stably transfected with pVHL_19_, or infected with control pSuperRetro retroviruses or with retroviruses directing expression of short hairpin RNA's (shRNA) targeting HIF-2α [35] that were provided by Dr. William Kaelin (Dana Farber Cancer Center), have been described previously [47]. For growth on collagen I, cells were grown on commercially prepared culture plates coated with a thin layer of collagen I (Becton Dickinson).

### Generation of VHL mutants

Mutant VHL sequences were either created *de novo *by sequential PCR steps using overlapping primers containing the desired point mutation, as previously described [11,52], or PCR amplified from existing VHL mutant constructs [11,53]. In creating pVHL_30 _expression constructs, mutant and wild-type (WT) VHL sequences from existing constructs or from PCR products (produced in the first step of *de novo *sequential PCR) were PCR amplified using the forward primer 5'GCGCGCGGATCCGCCACCATGCCCCGGAGGGCGGAGAACTGGGAGC 3' and reverse primer 5' CCCGGGCTCGAGTCAATCTCCCATCCGTTGATGTGCAATGC 3' (underlined sequences represent *BamH*I and *Xho*I restriction sites respectively). Wild-type and mutant pVHL_19 _sequences were similarly created, except the forward primer 5'GCGCGCGGATCCGCCACCATGGAGGCCGGGCGGCCGCGGCCCGTGC 3' was used in PCR amplifications.

### Retroviral expression constructs

PCR products containing wild-type (WT) and mutant pVHL_30 _coding sequences (as described above) were directionally cloned into the *BamH*I/*Xho*I sites in a pBabe-derived retroviral expression construct [54]. An empty plasmid (same pBabe-derived retroviral expression construct containing no VHL insert) was used as a control. PCR products containing wild-type and mutant pVHL_19 _coding sequences were cloned into *BamH*I/*Xho*I sites that were engineered into another retroviral vector, pQCXIP (Clontech, Mountain View, CA). The empty pQCXIP vector was used as a control for this series of mutant plasmids. To create a shRNA construct targeting p27, the oligonucleotides gatccccTGGTGATCACTCCAGGTAGttcaagagaCTACCTGGAGTGATCACCAtttttggaaa and agcttttccaaaaaTGGTGATCACTCCAGGTAGtctcttgaaCTACCTGGAGTGATCACCAggg (capital letters indicate RNA interference target sequences) were annealed and ligated into the *Bgl*II/*Hind*III sites in the vector pSuperRetro [55]. A shRNA construct targeting luciferase has been previously described [54]. All constructs were confirmed by DNA sequencing and/or restriction enzyme digestion.

### Transfections, Retroviral Production and Infection

To produce retroviral supernatants, each retroviral vector was co-transfected with the retroviral packing plasmid, pCL-Ampho [56], into 293T cells in 35 mm dishes using Lipofectamine Plus reagent (Invitrogen) as directed by the manufacturer. 293T cells from each transfection were replated into 60-mm dishes. Medium containing retroviruses was collected at 72 h post-transfection and filtered through a 45 μm pore-size filter. To create stable pools of retrovirally infected cells, parental RCC10 and A498 cells were incubated overnight in a mixture (1:1) of retroviral supernatant and fresh medium supplemented with polybrene (10 μg/ml). Three days later, cells were selected with puromycin (0.5 μg/ml) for 10 to 14 days. For stable pools of cells transfected with VHL expression plasmids, cells were transfected in 35 mm dishes using Lipofectamine Plus reagent. Cells were replated in 60-mm dishes (performed 2–16 hours post transfection), selected three days post-transfection with puromycin (0.5 μg/ml) for 10 to 14 days, and surviving cells were pooled.

### Western blotting

For all immunoblots, cells were grown to 100% confluency. 60 mm culture plates were rinsed with phosphate buffer saline (PBS) and cells were lysed using 150 to 175 μl of lysis buffer (50 mM HEPES (pH 7.6), 250 mM NaCl, 0.5% Nonidet P-40, 0.5% Triton X-100, 5 mM EDTA, 1 mM phenylmethylsulfonyl fluoride (PMSF), 1 mM Na_2_VO_3 _and 2 μg/ml each of aprotinin, bestatin, and leupeptin), incubating at 4°C for 30 minutes. Lysed cells were scraped with a plastic scraper, resuspended by pipetting, collected and then microcentrifuged for 15 minutes. Supernatants containing clarified protein lysates were removed and protein levels were determined by Bradford assay (Bio-Rad). Equal amounts (25–50 μg) of protein lysates were added to an appropriate amount of 2× SDS PAGE loading buffer and resolved by SDS-polyacrylamide gel electrophoresis and subsequently transferred to a polyvinylidene difluoride (PVDF) membrane overnight. Quantification of bands on western blots (using the antibodies below) was performed with Image J software (version 1.39 u).

### Antibodies

Rabbit polyclonal anti-HIF-2α antibody was from Novus Biological. Rabbit anti-GLUT-1 antiserum was from Alpha Diagnostics. Anti-VHL mAb 11E12 has been previously described [11]. Integrin and p27 mAbs were obtained from BD Transduction Laboratories. Rabbit Cyclin D1 antibody was from Santa Cruz Biotechnology. Rabbit anti-ZO-1 (Mid) was from Zymed. Anti-alpha tubulin mAb, as well as HRP-conjugated rabbit and mouse secondary antibodies were from Sigma. Texas red-conjugated goat anti-rabbit secondary antibody was from SouthernBiotech.

### Phase and Immunofluorescence Microscopy

For phase-contrast analysis of cell morphology, cells were grown on 60-mm collagen I coated plates until confluence and viewed on an Axiovert S100 microscope, with images captured by a Nikon DS-Fi1 CCD digital camera. For immunofluorescence experiments, cells were grown on coverslips in 6-well plates until they reached confluence. Cells were rinsed with PBS and then fixed and permeabilized by 1 min incubation in methanol/acetone (1:1) at -20°C and then rehydrated/washed twice for 3 min with PBS. Coverslips were incubated with blocking solution (1% goat serum in PBS containing 0.1% Tween-20) for 1 hr and then incubated with anti-ZO-1 antibody diluted 1:90 in blocking solution for 1 hr. Coverslips were washed (with rocking) 3 times for 5 min each with PBS containing 0.1% Tween-20 and then incubated for 1 hr with Texas Red-conjugated secondary antibody diluted 1:100 in blocking solution. Cells were washed 3 times as previously except that during the second wash, 4',6-diamidino-2-phenylindole (DAPI) was added at a concentration of 0.25 μg/ml. Cells were rinsed with PBS, mounted on slides with GelMount, and viewed at a magnification of 1000× on a Zeiss Axioskop microscope, with images collected by a Spot Insight QE digital camera. Random fields containing approximately equal numbers of nuclei (to eliminate any possible effects of confluency) were selected for viewing and exposures during imaging were kept constant for all samples (14 sec and 2 sec for ZO-1 and DAPI, respectively) to allow for more direct comparisons.

### Cycloheximide VHL Stability Assay

Each cell line was grown to confluence in 3 wells of 6-well culture dishes. Cells were then either left untreated or treated with cycloheximide (100 μg/ml; Sigma) for various time points and then lysed and assayed by western blotting as described previously.

## Results

### Type 1 VHL mutants have upregulated HIF-2α and downregulated p27

**Figure 1:**
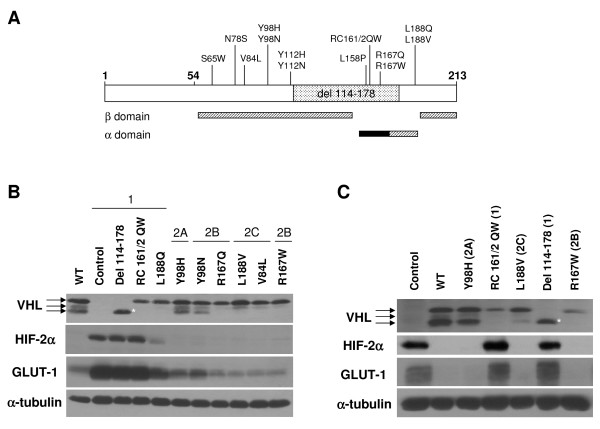
**Type 1, but not type 2 VHL mutants are grossly deficient in HIF-2α regulation**. (A) Diagrammatic representation of pVHL, with first and last amino acid residues in bold. Codon 54 represents the start of the pVHL_19 _product. Mutations used in this study and location of the α and β domains in the reported VHL structure [84] are shown. The location of the elongin binding domain is demarcated with a black box. (B) RCC10 VHL-null cells were stably infected with retroviruses produced with the empty vector with no VHL insert (control) or with wild-type (WT) or mutant pVHL_30 _expression constructs, as indicated. Cells were grown to confluence and prepared cell lysates were equally loaded and separated by SDS-PAGE. Western blots were performed for VHL, HIF-2α, and GLUT-1. Note that the 3 bands observed for VHL are closely-migrating isoforms of pVHL_30 _[11], but are not pVHL_19_, which was not produced by these constructs (data not shown). α-tubulin was also assayed to demonstrate equal loading. *indicates decreased size of VHL deletion protein. (C) A498 VHL-null cells were similarly stably infected and the resulting cell lines were analyzed by western blotting.

In order to investigate the cellular effects of specific VHL mutations, a set of mutant (and wild type) VHL retroviral expression constructs was created. Naturally occurring disease-associated mutations were chosen to represent all of the VHL disease subtypes based on information in the VHL mutation database [57], with 2 larger-scale rearrangements that were previously demonstrated to have a more complete inactivation of VHL function [11,53]. Note that while most of the mutations utilized here have been described in multiple VHL families, a few of the mutations (used here and in other reports) are not especially common and have been classified based on a single family. A list of all VHL mutations employed in this study (with references) can be found in Table 1 and are represented diagrammatically in Figure 1A. (References indicated in Table 1 are [4,11,28,29,53,58-67]). The mutant VHL expression constructs were used to generate several panels of cell lines that are described throughout this report.

Initially, VHL-negative RCC10 and A498 cells were infected with retroviruses directing expression of either wild type or mutant pVHL_30 _proteins or as a control, an empty vector retrovirus. In RCC10 cells (Figure 1B), type 1 VHL mutants were represented by constructs coding for pVHL with a deletion of amino acid residues 114 to 178 (del 114–178), a double point mutant RC161/2QW, the L188Q mutation, as well as control cells infected with a retrovirus lacking any VHL sequence. Type 2 VHL mutations included type 2A (Y98H), type 2B (Y98N, R167W, and R167Q), and type 2C mutations (L188V and V84L). A subset of these constructs was similarly infected into A498 cells (Figure 1C). For each infection, a stable pool (hereafter referred to as a cell line) was generated after puromycin selection. Western blots were performed to verify pVHL expression (Figure 1B and 1C, top panels) and HIF-2α levels (Figure 1B and 1C, second panels) in these cell lines. Cells expressing the type 1 VHL mutations (control, del 114–178, RC161/2QW, and L188Q) expressed high or intermediate levels of HIF-2α. However, low levels of HIF-2α similar to those observed with wild-type VHL were seen with all of the type 2 mutations (Y98H, Y98N, R167Q, R167W, L188V, and V84L) in this system. Levels of glucose transporter 1 (GLUT-1), a transcriptional target of HIF, were concomitantly upregulated in cell lines with type 1 VHL mutant proteins (Figure 1B and 1C, third panels).

**Figure 2:**
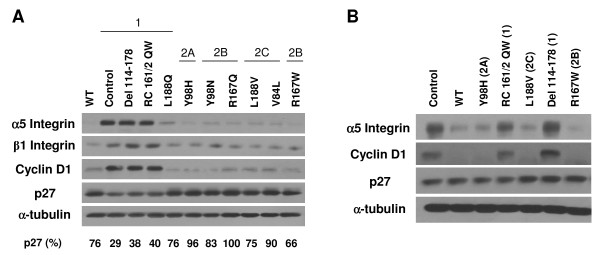
**Differential regulation of key markers of VHL function by type 1 and type 2 VHL mutants**. (A) Indicated RCC10 cell lines were grown to confluence on collagen I (under standard serum conditions) and prepared cell lysates were equally loaded and separated by SDS-PAGE. Western blots were performed for α5 and β1 integrins, cyclin D1, p27, and α-tubulin. VHL subtypes that mutations represent are provided at the top. Quantification of the band intensities for p27 blot (as a percent of the band with greatest intensity) is provided at the bottom. (B) A498 cells lines were similarly grown and analyzed by western blotting. VHL subtypes that mutations represent are provided in parentheses.

Several proteins were assayed as markers of VHL cellular functions (Figure 2A and 2B). For these analyses, cells were grown to confluence on collagen I because VHL-dependent effects on cell cycle and morphology have been shown to be more apparent under these conditions, even with standard serum levels in the culture medium [39,42,47]. Integrins are cell-extracellular matrix (ECM) adhesion proteins that are associated with cell morphological changes, are often upregulated in RCC cells that are more motile and invasive, and have been shown to be downregulated by pVHL in an HIF-independent manner [42,47,48]. In our cell lines, type 1 VHL mutants (control, del 114–178, and RC161/2QW, and to a lesser extent L188Q) expressed higher levels of α5 integrin (Figure 2A and 2B, top panels). In the RCC10 cell lines, β1 integrin levels were somewhat less variable, however the general trend of higher expression in type 1 mutant cells was upheld (Figure 2A, second panel). In contrast, wild-type and all type 2 VHL mutant cell lines had lower levels of these integrins (Figure 2A, top and second panels and Figure 2B, top panel).

Previous studies had indicated that pVHL expression affects levels of levels of cyclin D1 and the cyclin-dependent kinase (cdk) inhibitor, p27 [68-71]. Thus, the cell cycle status in our panels of mutant cells grown on collagen I was analyzed via western blots for cyclin D1 (Figure 2A, third panel and Figure 2B, second panel) and p27 (Figure 2A, fourth panel and Figure 2B, third panel). Type 1 mutant cells that had high HIF-2α levels (control, del 114–178, and RC161/2QW) expressed high levels of cyclin D1, indicating more active cell cycling. All other cell types, including all containing type 2 VHL mutants, demonstrated low levels of cyclin D1 equivalent to cells with wild-type VHL, suggesting proper pVHL functioning in regulating cell cycle exit. In RCC10, type 1 control, del 114–178, and RC161/2QW cells also displayed low levels of p27 expression, which correlated to their high levels of cyclin D1 and further signifies a deregulation of cell cycle exit in these cells. Again, all other RCC10 cells (including all type 2 VHL mutants) showed normal expression of p27 as compared to those with wild-type VHL, demonstrating their ability to properly exit the cell cycle. Interestingly, the type 1 mutant L188Q, which had only slightly elevated levels of HIF-2α, demonstrated neither cyclin D1 upregulation nor downregulation of p27, suggesting that either only high HIF-2α levels are associated with these phenomenon or that they are independent of HIF-2α status. Surprisingly, the A498 cell line did not behave like RCC10 cells, with no stark differences in p27 levels seen among cell lines expressing wild-type or mutant VHL proteins (Figure 2B, third panel). This result may be due to differences in the set of acquired mutations that these cells have gained following VHL loss or due to other intrinsic variations among these cells. Importantly, the lack of p27 effect on A498 cells occurred in spite of differences in HIF-2α regulation among the cell types, indicating that p27 expression is not likely to be directly regulated by HIF.

### Knockdown of p27 disrupts epithelial morphology and tight junction formation

**Figure 3:**
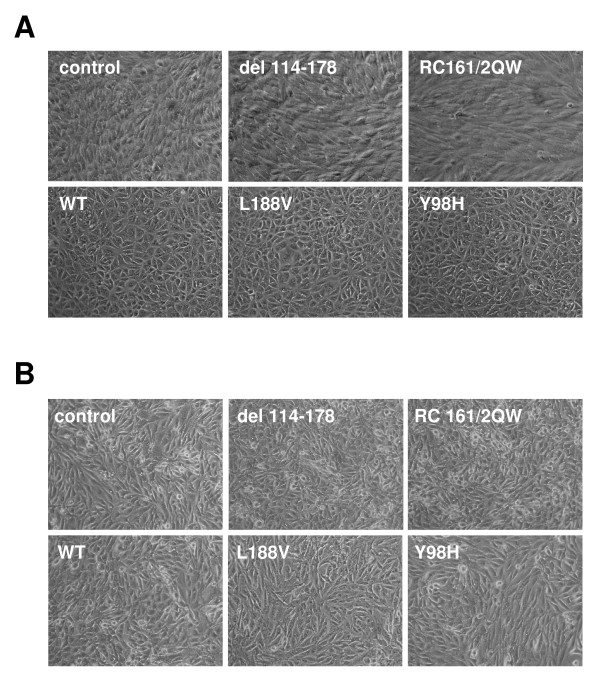
**RCC10 cells with type 1, but not type 2 mutant VHL proteins lack an epithelial-like morphology**. (A) Indicated RCC10 cell lines were grown to confluence on collagen I and photographed by digital phase microscopy (original magnification of 100×). Note the disorganized morphology of the control and the VHL del 114–178 and RC161/2QW-expressing cells. (B) A498 cell lines were similarly analyzed by phase microscopy. Note that all of the A498 cell lines display a disorganized non-epithelial phenotype.

Since the type 1 VHL mutant cell lines were strikingly different than the type 2 lines with respect to HIF-2α, GLUT-1, integrin, cyclin D1, and (in RCC10) p27 levels, we looked at whether there was any correlation between the levels of these proteins and the morphology of these cells. Images of the cells were captured upon confluent growth on collagen I. In RCC10, type 1 VHL cell lines that had high HIF-2α and low cyclin p27 (control, del 114–178, and RC161/2QW) had a more disorganized, fibroblastic phenotype whereas wild-type VHL and all of the type 2 cell lines displayed a more organized epithelial-like morphology (Figure 3A and Table 2). In line with the lack of effect of VHL expression on p27 levels in A498 (as seen in Figure 2B), VHL expression had no effect on the morphology of A498 cells, with VHL-negative, wild-type VHL, and all VHL mutant cell lines demonstrating a disorganized fibroblastic phenotype (Figure 3B). Thus, proper VHL-mediated upregulation of p27 (or conversely, downregulation of p27 due to acute VHL loss) appears to be associated with cell morphological changes.

**Table 2:** Summary of analyses of RCC10 and 786-O cell lines.

Cell Line	Subtype	Epithelial Morphology^a^		VHL Instability	Aberrant ZO-1
		RCC10	786-O		
Wild-type	-	+	+	-	-
control	1	-	-		+++
del 114–178	1	-	-	+^b^	++
RC161/2QW	1	-	-	+^b^	+++
S65W	1	-	N/D	+	++
N78S	1	-	N/D	+	++
L158P	1	-	N/D	+	+++
L188Q	1	+/-	+	+	+++

Y98H	2A	+	+	-	-
Y112H	2A	+	+	-	N/D

Y98N	2B	+	+	+/-	+
Y112N	2B	+	+	-	N/D
R167Q	2B	+	+/-	+	++
R167W	2B	+	+	+^b^	N/D

V84L	2C	+	+	N/D	N/D
L188V	2C	+	+	-	-

^a ^– indicates a disorganized fibroblast-like morphology

+/- indicates that an intermediate morphology was observed

^b ^previously demonstrated in [53]

N/D = not determined

**Figure 4:**
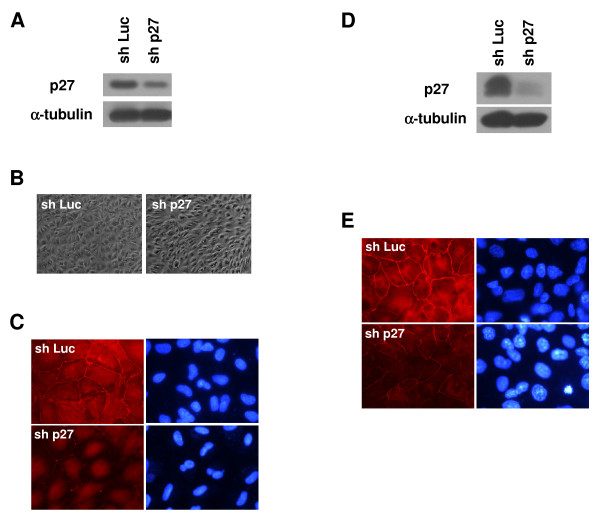
**Knockdown of p27 disrupts VHL-mediated tight junction formation**. (A) RCC10 cells stably expressing WT pVHL_30 _were infected with retroviruses containing shRNA constructs directed at luciferase as control (sh Luc) or p27 (sh p27). Approximately 2 weeks post-infection, cell lysates were equally loaded and subjected to p27 and α-tubulin western blotting. (B) RCC10 WT VHL cells infected with shRNA to luciferase or p27 were grown to confluence on collagen I and photographed by digital phase microscopy (original magnification of 100×). (C) RCC10 WT VHL cells infected with shRNA to luciferase or p27 were grown to confluence on coverslips and immunostaining for ZO-1 (left panels) was performed (original magnification of 1000×). DAPI labeled nuclei of corresponding cells are also shown (right panels). (D and E) 786-O cells stably expressing pVHL_19 _were infected and analyzed as in (A) and (C).

To further explore the relationship of p27 and cell morphology, a retroviral short hairpin RNA construct (shRNA) targeting p27 was created and used to infect the wild-type pVHL_30_-expressing RCC10 cell line. In comparison to control infected cells (infected with a shRNA targeting the non-human protein luciferase), the p27 shRNA efficiently knocked down p27 levels in these wild-type VHL cells (Figure 4A) and moreover, disrupted the epithelial morphology of these cells (Figure 4B).

Intracellular junctions such as adherens and tight junctions are known to greatly influence cellular morphology, and loss of both of these junctions is a characteristic of the epithelial-to-mesenchymal transition that accompanies tumor formation [72]. While a clear role of VHL in maintenance of adherens junctions has been established [50,73], VHL has also been previously shown to regulate tight junction complexes [44,74]. To determine whether the morphological effect that we observed with p27 knockdown occurs via disruption of tight junctions, fluorescent immunostaining of ZO-1, an important member of tight junction complexes, was performed. Whereas the control infected wild-type VHL cells showed ZO-1 staining in regions of cell-cell contact, forming rings of ZO-1 indicative of well-polarized cells, knockdown of p27 had a drastic effect on ZO-1 localization, with very little ZO-1 seen at cell-cell junctions (Figure 4C). These data indicate that regulation of p27 by VHL in RCC10 cells is necessary for proper tight junction formation.

To assess whether this phenomenon occurs in other VHL cell systems, we repeated the p27 knockdown using 786-O cells (a VHL-negative RCC cell line) in which the pVHL_19 _protein was stably reintroduced and expressed [11,47]. Knockdown of p27 in these cells led to diminution of ZO-1 staining (Figure 4D and 4E), albeit not to the extent seen with p27 knockdown in pVHL_30_-containing RCC10 cells. The reason for the difference between cell lines is not clear and suggests that p27 levels influence, but perhaps are not the sole determinant of tight junction formation in VHL-positive RCC cells.

### Lack of difference in cell morphology among type 2 VHL mutants is not due to the mechanism of VHL reintroduction

**Figure 5:**
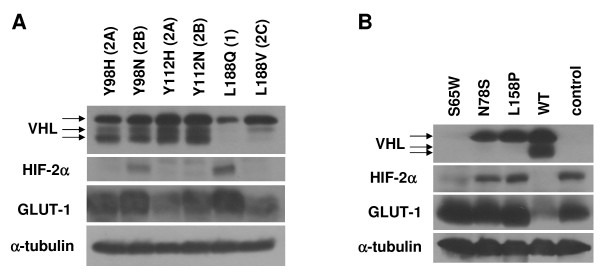
**Additional RCC10 cell lines were generated**. (A) RCC10 VHL-null cells were stably transfected with pVHL_30 _expression constructs. Cells were grown to confluence and prepared cell lysates were equally loaded and separated by SDS-PAGE. Western blots were performed for VHL, HIF-2α, and GLUT-1. α-tubulin was also assayed to demonstrate equal loading. (B) RCC10 VHL-null cells were stably infected with retroviruses containing mutant pVHL_30 _expression constructs (S65W, N78S, L158P) and assayed along with original control and WT VHL RCC10 cells as in (B).

While our data indicated that type 1 VHL mutants differ from type 2 mutants, especially with respect to cell morphology, we had seen very little difference among the type 2 mutations. Since previous studies had indicated that type 2B VHL mutants are more defective in HIF-α regulation than type 2A mutants [30,34], we wished to determine whether the retroviral infections that we utilized, which can introduce multiple copies of the expression construct, might be influencing our results. Toward this end, we transfected RCC10 cells with our pVHL_30 _constructs, selected with puromycin, and generated pools of stable transfectants (Figure 5A). For these experiments, we utilized paired mutations with alternate amino acids at the same VHL codon that result in different VHL disease subtypes, based on the notion that they may reveal differences in VHL biochemical functions that are relevant to differential tumor risk. While upregulated HIF-2α and GLUT-1 were seen with type 1 mutant L188Q and with the type 2B mutant Y98N (as compared to wild-type VHL and type 2A mutant lines), the type 2B mutant Y112N cell line did not demonstrate this effect, suggesting that the currently accepted division of VHL subtypes does not perfectly conform to the status of HIF-α regulation. Of note, all of the transfected type 2 VHL cell lines demonstrated an epithelial morphology including the type 2B mutants (Table 2), indicating that method of VHL reintroduction was not a factor in this phenotype. However, our data leaves open the possibility that very high levels of HIF-2α (as seen with almost all of the type 1 VHL mutants) are associated with a lack of epithelial morphology.

### Type 1 mutant VHL proteins are unstable

Since the majority of the type 1 VHL mutations utilized so far in our assays were major alterations in pVHL, additional type 1 VHL mutations, all missense (S65W, N78S, and L158P), were studied (Figure 5B). The new RCC10 mutant cell lines were generated by retroviral infection as previously. All of these additional type 1 VHL mutant RCC10 cell lines showed upregulated HIF-2α and GLUT-1, although the S65W mutant had slightly lower, but still upregulated HIF-2α. As expected, all of the new missense type 1 VHL mutant cell lines displayed the same disorganized, fibroblastic morphology as seen with the other type 1 VHL mutants (Table 2).

**Figure 6:**
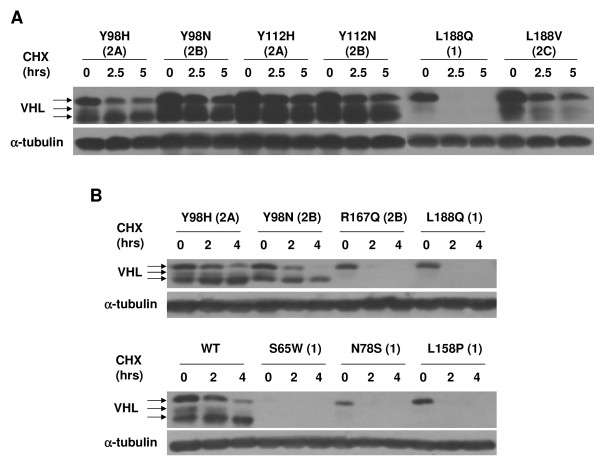
**Type 1 VHL mutant proteins are unstable**. (A) Indicated stably transfected RCC10 cell lines were incubated in media with no cycloheximide (0 time point) or with cycloheximide (100 μg/ml) for the indicated lengths of time. Cell lysates were prepared, equally loaded and subjected to VHL and α-tubulin western blotting. (B) Cycloheximide stability assay as in (A) was performed on the indicated stably infected RCC10 cell lines.

The S65W mutant cell line was interesting in that the level of VHL protein was considerably lower than for all other mutants. Since the 5' regions were identical for all of the expression constructs used, this finding suggested that there are differences in the stability of the various VHL mutants. In fact, many of the type 1 VHL mutants expressed in these studies showed slightly lower levels of pVHL expression (see Figures 1B, and 1C, and Figure 5A). To further investigate, a cycloheximide stability assay was performed, focusing on the type 1 VHL missense mutations. RCC10 cell lines were treated with cycloheximide for given amounts of time and levels of VHL protein were assayed by western blotting (Figure 6). All of the type 1 missense mutants tested (L188Q, S65W, N78S, and L158P) were unstable, with no detectable protein seen after 2 hours (or 2.5 hours in the case of L188Q) of cycloheximide. Since both transfected and infected cell lines were utilized (Figures 6A and 6B), this effect was also independent of the mechanism in which VHL was replaced in these cells. In accord with a previous observation that elongin B and C binding to pVHL confers stability and that mutations in the elongin binding domain render VHL proteins unstable [53], the type 2B mutant R167Q was relatively unstable in the assay reported here, with slightly detectable levels of VHL protein after 2 hours of cycloheximide treatment. However, other type 2B VHL mutations containing mutations outside of the elongin binding domain (Y98N and Y112N) were much more stable, indicating that the instability of the R167Q mutant is not indicative of the entire 2B VHL subtype. We also noted that the abundance of faster migrating pVHL_30 _isoforms for any particular VHL mutant (see bottom arrows for VHL in Figure 6) was a predictor of VHL stability, since those VHL mutant proteins that produced lower levels of these faster migrating isoforms (type 1 mutations and R167Q) were the least stable in our assay. The significance of this finding is currently unclear, but may have functional implications.

### Lack of morphological change by type 1 VHL mutations is recapitulated in 786-O cells

**Figure 7:**
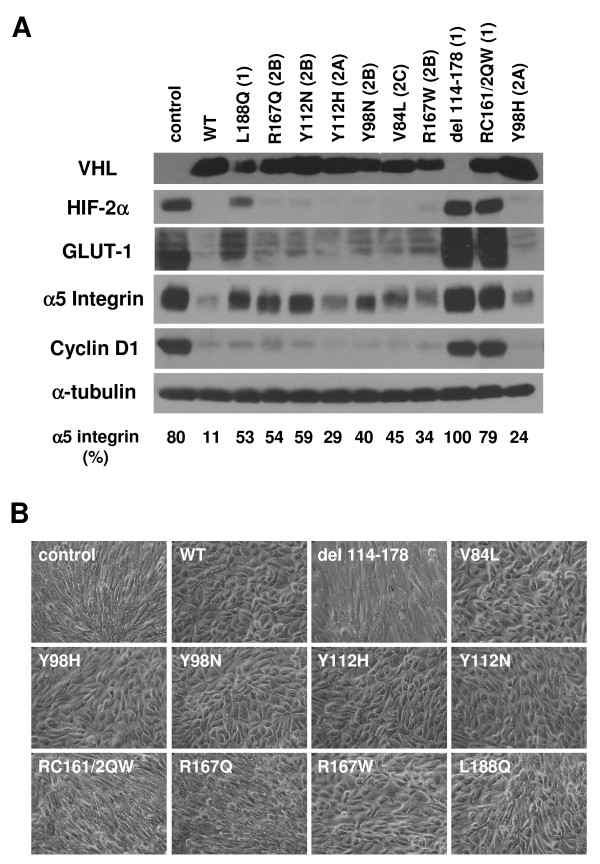
**Differential regulation of key markers of VHL function by type 1 and type 2 pVHL_19 _mutants in 786-O**. (A) 786-O cells were stably transfected with empty vector control or wild-type (WT) or mutant pVHL_19 _expression constructs, as indicated. Cells were grown to confluence on collagen I and prepared cell lysates were equally loaded and separated by SDS-PAGE. Western blots were performed for VHL, HIF-2α, GLUT-1, α5 integrin, cyclin D1, and α-tubulin. A faster migrating band is seen for del 114–178 upon darker exposure of the VHL blot (not shown). Quantification of the band intensities for the α5 integrin blot (as a percent of the band with greatest intensity) is provided at the bottom. (B) Indicated 786-O stable cell pools were grown to confluence on collagen I and photographed by digital phase microscopy (original magnification of 100×). Note the disorganized morphology of the control and the VHL del 114–178 and RC161/2QW-expressing cells.

Since the results of morphological analyses performed thus far in this study had relied on the RCC10 cell line, we wished to extend these findings to another VHL-negative cell line, 786-O. We generated a panel of 786-O cells as previously, with the following exceptions: 1) VHL and control expression constructs were transfected rather than selected into these cells; 2) pVHL_19 _expression constructs were used (rather than pVHL_30_); and 3) a different expression vector was utilized (see Methods). Analysis of proteins associated with VHL function was similar to those seen with pVHL_30 _in RCC10 cells, with upregulated HIF-2α, GLUT-1, and cyclin D1 detected in most of the type 1 VHL mutant lines (control, RC161/2QW and del 114–178) and partial upregulation seen with L188Q (Figure 7A). Differences in p27 abundance in 786-O were much more subtle and only partially recapitulated the pattern seen in RCC10 (data not shown). Note that PTEN is inactivated in 786-O [75], which is likely to affect p27 regulation in this cell line.

In contrast to the other markers studied, α5 integrin levels seemed to correlate closely with VHL disease subtype in 786-O cells, with the lowest levels seen with wild-type VHL, slightly elevated levels seen with type 2A and 2C VHL mutants (2A: Y98H, Y112H; 2C: V84L), higher levels seen with a majority of the type 2B VHL mutants (R167Q, Y112N, Y98N) and still higher levels in the type 1 VHL mutants. Cell morphological analyses in 786-O yielded very similar to results as in RCC10, with type 1 control, RC161/2QW and del 114–178 cell lines demonstrating a fibroblastic, disorganized phenotype and all other cells displaying an epithelial phenotype, with the exception of the R167Q mutant, which had an intermediate morphology (Figure 7B). The L188Q mutant, which had shown an intermediate phenotype in RCC10, was seen to be somewhat more epithelial in 786-O. The results of the morphological analyses with both RCC10 and 786-O cell lines are summarized in Table 2.

### Tight junctions are severely compromised in type 1 VHL mutants

**Figure 8:**
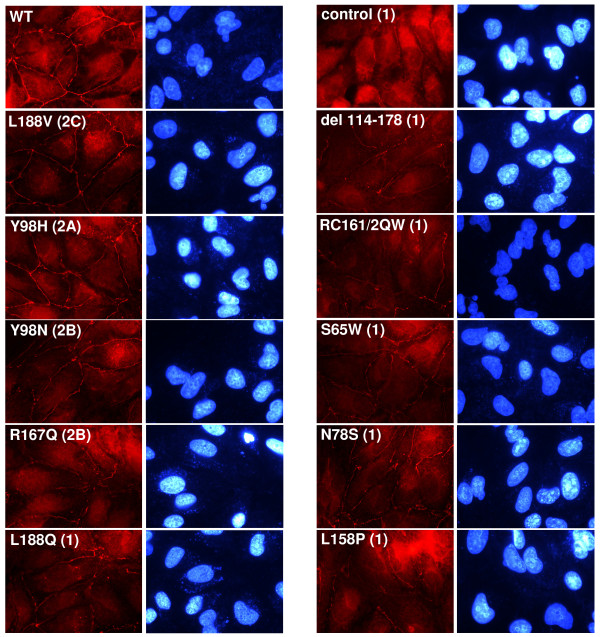
**Contrasting regulation of tight junctions among VHL mutants of different disease types in RCC10**. RCC10 cells lines (as indicated; all created by retroviral infection) were grown to confluence on coverslips and immunostaining for ZO-1 (left panels) was performed (original magnification of 1000×). DAPI labeled nuclei of corresponding cells are also shown (right panels).

We next wanted to look at differences in the formation of tight junctions among the VHL mutant cell lines. The panel of RCC10 cells was immunostained for ZO-1 (Figure 8). Cells with wild type or type 2C or 2A VHL mutants (L188V or Y98H, respectively) demonstrated adequate ZO-1 staining at all cell-cell borders. Some disorganized ZO-1 staining (e.g., entire borders not stained) was seen with the type 2B VHL mutants, Y98N and to a slightly greater extent, R167Q. An overall more disorganized ZO-1 staining pattern (with staining largely absent from the majority of cell-cell junctions) was seen with type 1 VHL mutants (L188Q, control, del 114–178, RC161/2QW, S65W, N78S, and L158P). The results of the tight junction analysis are summarized in Table 2.

Similar results were obtained using the 786-O cell lines (data not shown). In summary, proper formation of tight junctions appears to be severely compromised with VHL type 1 mutants and the R167Q type 2B mutant, somewhat aberrant with the Y98N type 2B mutant, and close to wild-type with type 2A and 2C mutants, and thus seems to loosely correspond with the risk of RCC associated with these VHL disease subtypes [30].

**Figure 9:**
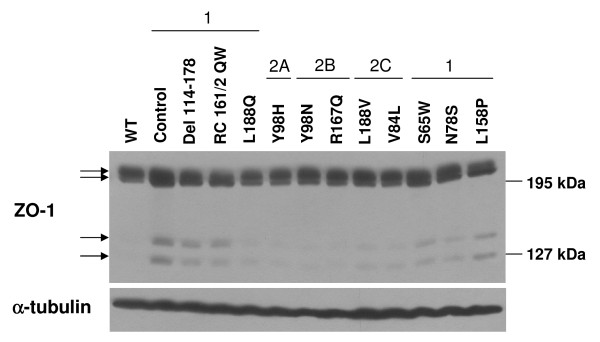
**Contrasting levels of ZO-1 fragments among VHL mutants of different disease types in RCC10**. RCC10 cells lines (as indicated; all created by retroviral infection) were grown to confluence. Cell lysates were prepared and equally loaded and separated by SDS-PAGE. Western blots were performed for ZO-1 and α-tubulin. Arrows indicate ZO-1 immunoreactive proteins.

To determine whether the VHL mutants varied in their overall levels of ZO-1, a western blot was performed using RCC10 lysates (Figure 9). ZO-1 levels were very slightly upregulated in most type 1 VHL mutants as compared to wild-type VHL-expressing cells. Moreover, two faster migrating bands, perhaps representing ZO-1 cleavage products, were detected at the greatest levels in type 1 VHL mutant lysates and were absent in cells with wild-type VHL. The significance of this observation is unclear, however the appearance of ZO-1 cleavage products in cells challenged with apoptotic stresses has been associated with impaired localization to tight junctions [76], which agrees with the ZO-1 mislocalization seen in these type 1 VHL mutant cell lines. Extending these findings, one possibility is that the presence of these faster migrating ZO-1 bands indicates that type 1 VHL mutants are in a heightened state of cellular and/or metabolic stress, which has been documented for VHL-negative cells [49,77].

### HIF-2α plays a partial role in regulation of tight junctions

**Figure 10:**
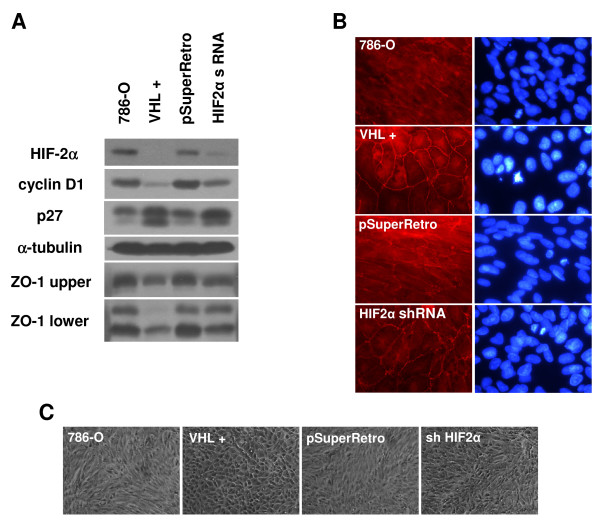
**Tight junctions are affected by levels of HIF-2α in 786-O**. (A) Parental 786-O cells (786-O), 786-O cells stably transfected with VHL (VHL +), and 786-O cells infected with an empty retrovirus (pSuperRetro) or infected with HIF-2α shRNAs (HIF2α shRNA), as described in [47], were grown for 1 week past confluence on collagen I coated culture dishes. Cell lysates were prepared, equally loaded, and western blotted for HIF-2α, cyclin D1, p27, α-tubulin and ZO-1 (both upper and lower immunoreactive species that were seen in Figure 9). (B) Indicated cell lines that were assayed in (A) were grown to confluence on coverslips and immunostained for ZO-1 (left panels; original magnification of 1000×). DAPI labeled nuclei of corresponding cells are also shown (right panels). (C) Indicated cell lines that were assayed in (A) and (B) were grown to confluence on collagen I and photographed by digital phase microscopy (original magnification of 100×).

Our data had indicated that type 1 VHL mutants with high levels of HIF-2α have dysregulated tight junctions. To examine whether a direct link between HIF-α levels and ZO-1 localization exists, we turned to a 786-O cell line in which HIF-2α levels had been lowered by shRNA vectors [47]. Note that 786-O cells have been reported to only express the HIF-2α isoform [20]. We had previously compared HIF-2α knockdown cells to parental cells, control infected cells, and those in which VHL had been stably reintroduced [47]. In that study, it had been determined that although regulation of cell cycle progression is restored by HIF-2α knockdown, the morphology of the cells was not restored to the full epithelial phenotype seen with VHL re-expression [47]. In the current studies, a western blot for proteins associated with pVHL function was performed (Figure 10). As previously, a decrease in HIF-2α levels comparable to that seen with wild-type VHL expression was observed with the shRNA (Figure 10A, top panel). In agreement with prior cell cycle findings, HIF-2α knockdown led to cyclin D1 downregulation and upregulation of p27, also comparable to the effects of wild-type VHL expression in these cells (Figure 10A, second and third panels). Importantly, ZO-1 levels were slightly lower in both wild-type VHL and HIF-2α knockdown cells (Figure 10A, second panel from the bottom), suggesting that the minor differences in ZO-1 levels seen between cell lines might be regulated by HIF-2α levels. Also, levels of the potentially cleaved faster-migrating ZO-1 products were greater in the parental and control infected cells than in cells with wild-type VHL or HIF-2α shRNA (Figure 10A, bottom panel), also suggesting that HIF-2α influences these ZO-1 fragments. However, levels of these fragments were slightly higher in HIF-2α knockdown cells than those with wild-type VHL, indicating that formation of this ZO-1 fragment may be only partially regulated by HIF-2α.

We performed ZO-1 immunofluorescence with these cell lines, to see if the differences in ZO-1 protein levels affect tight junction formation (Figure 10B). The ZO-1 staining pattern closely mirrored the western blot analysis: both parental and control infected cells showed a disorganized ZO-1 pattern, while cells with wild-type VHL displayed abundant ZO-1 staining at cell-cell borders. HIF-2α knockdown cells demonstrated partially restored ZO-1 staining, with more ZO-1 seen at cell-cell junctions in comparison to the negative controls, but with a slightly disorganized localization marked by incomplete and somewhat jagged staining at some of the cell borders. These results again indicate that tight junctions are partially regulated by HIF-2α.

To see whether the observed differences in ZO-1 staining are associated with changes in cell morphology, we captured phase-contrast images of these cells (Figure 10C). As expected, 786-O cells stably expressing wild-type VHL had a normal epithelial-like appearance marked by cobblestone shaped cells, while parental and control infected cells showed a disorganized, fibroblastic morphology. However, cells with HIF-2α knocked down also showed a mostly disorganized phenotype, in spite of the observed increase in ZO-1 staining at cell borders. Overall, these results suggest that while HIF-α downregulation contributes to tight junction formation, this outcome is not sufficient for cells to obtain the same epithelial morphology as seen when VHL is reintroduced into these cells.

## Discussion

In the present study, we have compared a panel of VHL mutants that cover the spectrum of VHL disease subtypes with respect to their ability to bring about the cell morphological changes that are observed with wild-type VHL in renal cells. We found that type 1 VHL mutants were vastly deficient in producing an epithelial-like morphology and higher levels of HIF-2α (and perhaps cyclin D1) are likely to impact this phenotype. The disorganized morphology of type 1 mutants was concomitant with a lack of VHL protein stability and higher levels α5 integrin (and for RCC10, β1 integrin). Moreover, the lack of epithelial morphology seen with type 1 VHL mutants was marked by aberrant localization of the tight junction marker, ZO-1. Lowering of HIF-α levels was seen to greatly influence tight junction formation, but was not sufficient to bring about a full restoration of epithelial morphology. The significance of these findings is further discussed below.

In this report, we found that type 1 VHL mutants had considerably elevated levels of HIF-2α. We analyzed VHL regulation of HIF-2α rather than HIF-1α because HIF-2α (but not HIF-1α) is expressed in all of the RCC cell lines utilized [20] and more importantly, HIF-2α is regarded as the key HIF-α isoform in RCC formation [78,79]. While type 1 VHL mutants were grossly deficient in their ability to cause degradation of HIF-2α, the majority of type 2 mutations in this study were as efficient as wild type VHL, regardless of their type 2 subtype. This result differs from previous studies in which type 2B mutants were seen to be deficient in HIF-2α ubiquitination [30,34]. The reasons for the differences among these studies are currently unclear and may relate to the levels of pVHL that result from exogenous expression, as overexpression may compensate for incomplete loss of pVHL function with some of the mutants but may not compensate for complete loss of function.

Type 1 VHL mutants, in addition to defects in regulation of HIF-2α, had lower levels of p27 in RCC10 cells as compared to cells with wild-type VHL or type 2 mutants. One exception was the reported type 1 mutant, L188Q, which did not demonstrate lower p27 levels (and was also seen to have intermediate phenotypes in a number of our assays). Since the classification of L188Q as a type 1 mutation is based on a single small family [29], it is possible that this designation is incorrect. Notably, the downregulation of p27 observed here with the other type 1 mutants is opposite from a study in mouse embryo fibroblasts in which loss of VHL led to p27 upregulation [80], which may indicate that VHL's influence on p27 is cell-type dependent. However, the decreased levels of p27 observed with our type 1 VHL mutants are likely to be clinically relevant since p27 is an effector of cell cycle withdrawal and cell differentiation and low levels of p27 are associated with poorly differentiated invasive tumors and a poor prognosis in many epithelial cancers [81,82]. Moreover, we observed that diminution of p27 levels disrupted tight junction formation and epithelial cell morphology (to a greater degree in RCC10 than 786-O). In RCC10 cells, high p27 levels correlated well with low HIF-2α and cyclin D1 levels, however VHL proteins that were able to properly regulate HIF-2α (including all type 2 VHL mutants) were unable to produce changes in p27 abundance in A498 cells, suggesting that p27 levels may be only indirectly affected by HIF transcriptional activity and that VHL might modulate p27 levels through some other mechanism. Given that p27 is regulated at the transcriptional, translational, and post-translational levels (reviewed in [83]), the effect of pVHL on p27 levels warrants further study.

Overall, the present findings indicate that the type 1 VHL mutations (including missense mutations) represent a more complete loss of VHL function as compared to the type 2 mutations. Likely related to this loss of function, proteins produced by type 1 VHL mutant cell lines were shown to be very unstable as compared to the type 2 mutants. A number of the type 1 missense mutations contain substitutions in amino acids whose side chains make key contacts in pVHL folding [84], which can cause gross misfolding or leave pVHL in an exposed, chaperonin-bound state [85]. Some of the type 1 VHL mutant proteins have also been shown to exhibit reduced binding to elongin C, which is important for pVHL stability [30,53]. It is entirely possible that other type 1 missense mutations, for which the relationship of the amino acid substitution to pVHL folding is unclear from the present 3-dimensional structure (such as S65W), will also show impaired folding and that this defect may underlie the more complete loss of function associated with type 1 mutations. Interestingly, one type 2B mutant, R167Q, was unstable yet retained normal HIF-2α regulation in our assays, signifying that deficiencies in stability and VHL function are not synonymous. Again, it is probable that overexpression of this mutant compensated for a partial defect in ubiquitination function at steady-state levels. However, the lack of stability observed for the R167Q mutant indicates that considerable amounts of new protein synthesis may be necessary to attain this steady-state level. At endogenous protein levels, a more noticeable, but not complete loss of HIF-α regulation is likely to be revealed for this mutant, as has been previously demonstrated [30,34]. It is important to point out that the notion that type 1 VHL mutants represent a greater loss of VHL function than the type 2B mutations can be viewed as somewhat inconsistent with the fairly equivalent high risk of RCC among types 1 and 2B. However, it is possible that in individuals with type 1 VHL mutations, higher levels of HIF-2α and of the proapoptotic HIF target gene, EglN3, cause some culling of renal cells upon loss of the wild type allele, in a manner similar to as has been suggested for pheochromocytoma [33]. Lending support to this hypothesis, a recent report showed that mouse ES cells homozygous for the type 2B R167Q mutation demonstrate a growth advantage over VHL-null ES cells (*i.e.*, a type 1 mutant) in a teratoma xenograft assay [86], with the HIF-2α profile of their cells paralleling those seen here. More *in vivo *studies comparing the tumorigenic events that occur with specific VHL mutants are necessary to fully understand their differential risks of RCC.

Although in our studies we did not observe major differences among the type 2 VHL mutants in RCC10 and 786-O with respect to HIF-2α levels, p27 levels (in RCC10), or cell morphological changes, there were some VHL-mediated activities that seemed to more closely correlate with VHL disease subtypes: localization of ZO-1 to cell-cell junctions and regulation of α5 integrin levels (in 786-O). There was a gradient in the degree of disruption of both of these functions (*i.e.*, absence of correct ZO-1 localization to tight junctions and high levels of α5 integrin), with type 1 VHL mutants being the most compromised, followed in order by type 2B and type 2A, whereas type 2C mutants behaved like wild-type. Thus, both α5 integrin levels (in 786-O) and lack of ZO-1 regulation by VHL parallel the relative risk of RCC associated with the VHL disease subtypes of specific mutations: higher risk in types 1 and 2B, low risk in type 2A and no risk in type 2C (reviewed in [2]). Although it is unclear whether integrin levels and ZO-1 localization are regulated through similar cellular pathways, it may be that cell-cell interactions and cell-matrix interactions are coordinately regulated in a VHL-dependent manner. It is worth noting that VHL activities independent of HIF-α ubiquitination are required for both proper ZO-1 localization (shown here) and regulation of α5 integrin levels [47,48]. One distinct possibility is that the observed VHL-mediated ZO-1 localization and/or integrin regulation is influenced by another reported substrate for VHL ubiquitination, atypical protein kinase C [87], for which roles in both tight junction formation and integrin expression and adhesion have been demonstrated [88-90]. However, HIF-α independent roles of pVHL in cytoskeletal microtubule stability and extracellular matrix formation [41,43] might also impinge on ZO-1 and integrin regulation.

Whereas there is well-documented evidence that the loss of HIF-α regulation upon VHL inactivation is critical for RCC progression in VHL disease [35-38], it is unclear whether HIF-α dysregulation is sufficient for the initiation of renal tumors and/or cysts. Based on the data presented in this report and elsewhere, we speculate that the proper control of integrins and tight junctions may be important for VHL tumor suppressor function at the early stages of tumor formation. Integrins play key roles in cell adhesion and migration, and can signal to pathways leading to cell proliferation, invasion and survival (reviewed in [91]). α5 integrin has been shown to be expressed in clear cell renal tumors and renal cysts, but not in normal proximal and distal tubules [92], lending some import to the increased α5 integrin levels seen here to correlate with VHL mutations that have a higher RCC risk and suggesting that α5 integrin upregulation might be an early step in tumor initiation. The loss of tight junctions and associated cell-polarity is also likely to be tumor promoting and might also be an early step in tumor initiation, leading to an ability of kidney epithelial cells to break free of cell-cell contact restraints and to proliferate. Note that while these studies reported here were being completed, Harten et al. demonstrated that tight junctions are disrupted in both RCC tumors and renal pre-malignant cystic lesions that develop following VHL loss, supporting this notion [74]. Decreased expression of the adherens junction protein, E-cadherin, following VHL loss [50,73] may further break down cell-cell adhesion, aiding in tumor formation. However, the results of this study indicate that some additional VHL function(s) may need to be lost for full epithelial to mesenchymal transition, as noted below.

The present data shows that although there are VHL functions independent of HIF-α regulation that impinge upon aspects of cell morphology, HIF-α levels still have a large influence on cellular morphological differentiation. Along these lines, hypoxic conditions have been shown to promote both an epithelial to mesenchymal switch and loss of ZO-1 localization at cell-cell junctions of primary renal tubule cells [51]. Here, high levels of HIF-2α correlated well an undifferentiated fibroblastic cellular phenotype. However, shRNA knockdown of HIF-2α levels in 786-O cells, which has been shown to correct deficiencies in E-cadherin expression [73] and here to promote ZO-1 localization to tight junctions, was not sufficient to revert the cells to an epithelial morphology. The reasons for this apparent paradox are not currently clear, but suggest that some additional pVHL activity is necessary to bring about the full epithelial morphology of cells. One remote possibility, based on the findings of Harten et al. that knockdown of HIF-1α restores tight junctions and cell morphology more efficiently than knockdown of HIF-2α [74], is that 786-O cells may express some residual HIF-1α that maintains the fibroblastic appearance. Nonetheless, other HIF-α independent pVHL functions, such as integrin regulation and fibronectin deposition [41,47,48,93] are likely to play roles mediating an epithelial phenotype, since the interaction between integrins and extracellular matrix does affect cell morphology (reviewed in [91]). While further study may be necessary to sort out the exact mechanisms by which pVHL regulates an epithelial morphology, the current data supports the supposition that both HIF-α dependent and HIF-α independent pVHL activities are necessary for the regulation of cell morphology.

## Conclusion

Expression of Type 1 VHL mutants in RCC10 and 786-O renal carcinoma cells failed to restore an epithelial morphology, unlike wild type VHL and the majority of type 2 VHL mutants. Type 1 VHL mutants exhibited elevated levels of HIF-2α, cyclin D1, α5 integrin, and lower levels of p27 (in RCC10), and demonstrated protein instability (which was also seen with the type 2B mutant R167Q). Tight junctions, as visualized by ZO-1 immunostaining, were most aberrant in type 1 VHL mutants, although tight junction disorganization was seen with type 2B mutants and to a lesser extent with type 2A mutants, but not with type 2C. Knockdown of HIF-2α partially restored tight junctions, but did not restore an epithelial morphology, suggesting that VHL activities independent of HIF-α ubiquitination are also needed for regulation of this cellular phenotype. In conclusion, the data presented here highlight several deficiencies in key effectors of cell cycle regulation and cell-cell and cell-matrix associations in VHL mutants (especially type 1), a combination of which are likely to contribute to the initiation of VHL-associated renal tumorigenesis.

## Abbreviations

(DAPI): 4',6-diamidino-2-phenylindole; (GLUT-1): glucose transporter 1; (HIF): hypoxia-inducible factor; (PBS): phosphate buffer saline; (RCC): renal cell carcinoma; (shRNA): short hairpin RNA; (VHL): von Hippel-Lindau; (pVHL): von Hippel-Lindau gene product; (WT): wild-type.

## Competing interests

The authors declare that they have no competing interests.

## Authors' contributions

VB, AR, AEA, and ARS were involved in the overall study design and coordination and performed many of the experimental procedures and data analyses. BI and TP performed some of the molecular cloning, cell culture, and western blot analyses. VB and AR wrote parts of the original draft of the manuscript, and ARS added parts and edited the manuscript. ARS conceived of the study and served as the principal investigator. All authors have read and approved the final manuscript.

## Pre-publication history

The pre-publication history for this paper can be accessed here:

http://www.biomedcentral.com/1471-2407/9/229/prepub
